# Giant refractometric sensitivity by combining extreme optical Vernier effect and modal interference

**DOI:** 10.1038/s41598-020-76324-7

**Published:** 2020-11-09

**Authors:** André D. Gomes, Jens Kobelke, Jörg Bierlich, Jan Dellith, Manfred Rothhardt, Hartmut Bartelt, Orlando Frazão

**Affiliations:** 1 Leibniz Institute of Photonic Technology (Leibniz-IPHT), Albert-Einstein-Strasse 9, 07745 Jena, Germany; 2 INESC TEC and Department of Physics and Astronomy, Faculty of Sciences, University of Porto, Rua do Campo Alegre 687, 4169-007 Porto, Portugal

**Keywords:** Fibre optics and optical communications, Optical sensors

## Abstract

The optical Vernier effect consists of overlapping responses of a sensing and a reference interferometer with slightly shifted interferometric frequencies. The beating modulation thus generated presents high magnified sensitivity and resolution compared to the sensing interferometer, if the two interferometers are slightly out of tune with each other. However, the outcome of such a condition is a large beating modulation, immeasurable by conventional detection systems due to practical limitations of the usable spectral range. We propose a method to surpass this limitation by using a few-mode sensing interferometer instead of a single-mode one. The overlap response of the different modes produces a measurable envelope, whilst preserving an extremely high magnification factor, an order of magnification higher than current state-of-the-art performances. Furthermore, we demonstrate the application of that method in the development of a giant sensitivity fibre refractometer with a sensitivity of around 500 µm/RIU (refractive index unit) and with a magnification factor over 850.

## Introduction

Recently, the use of the Vernier effect has found great interest in interferometric sensing concepts, and especially in fibre Fabry–Perot elements, as a tool to considerably increase their sensitivity^1–4^. This concept is based on the beating signal of two slightly detuned interferometers (one sensing interferometer and one reference interferometer)^5–7^. The beating signal produces an additional modulation, which can be observed in a limited free spectral range and with lower spectral resolution than for a single fundamental interferometer. The detuning of the two interferometers, however, is quite a tricky problem. To achieve high magnification factors (typically in the order of tens^7–10^), a small detuning value would be desirable. On the other hand, detuning by a very small amount (as an extreme optical Vernier effect) may result in a beating modulation with long period, which may become undetectable for a limited spectral range available. These contradictory requirements present a considerable challenge for the experimental implementation of such sensors with large magnification factors for sensitivity. Here we present a method to overcome this dilemma by using few modes instead of a single mode in the sensing interferometer (ideally two modes), preferably with a relatively large effective refractive index between them. The reference interferometer is in tune with the fundamental mode (mode #1) of the sensing interferometer, which would provide a huge magnification factor but with an extremely large immeasurable envelope. On the other hand, for a higher order mode (mode #2) it represents a lower magnification factor but with a smaller and measurable Vernier envelope, since the mode-effective refractive index is different, generating a Vernier effect slightly less in tune. However, when both responses superimpose, the Vernier envelope is still measurable, whilst maintaining a huge magnification factor typical for large immeasurable envelopes. Therefore, the method of combining the two modes in the sensing interferometer provides magnification factors an order of magnitude beyond the expected limits for the standard Vernier effect technique. Here we demonstrate such a result with a giant magnification factor by implementing a few-mode fibre-optic Fabry–Perot refractometer in combination with a single-mode Fabry–Perot reference interferometer, achieving a giant magnification factor of over 850 for the measurement of the liquid refractive index of aqueous solutions. Below we present at first the experimental configuration of the microstructured Fabry–Perot interferometers (FPIs). Then the case of a two-mode sensing interferometer (as a simplified first approach to the experimental case of a few-mode sensing interferometer) is simulated to show the properties of the effect. Lastly, the case of a single-mode and a few-mode sensing interferometer in combination with a reference interferometer for the Vernier effect will be analysed and discussed.

## Results

### Experimental setup, spectral control and analysis

**Figure 1:**
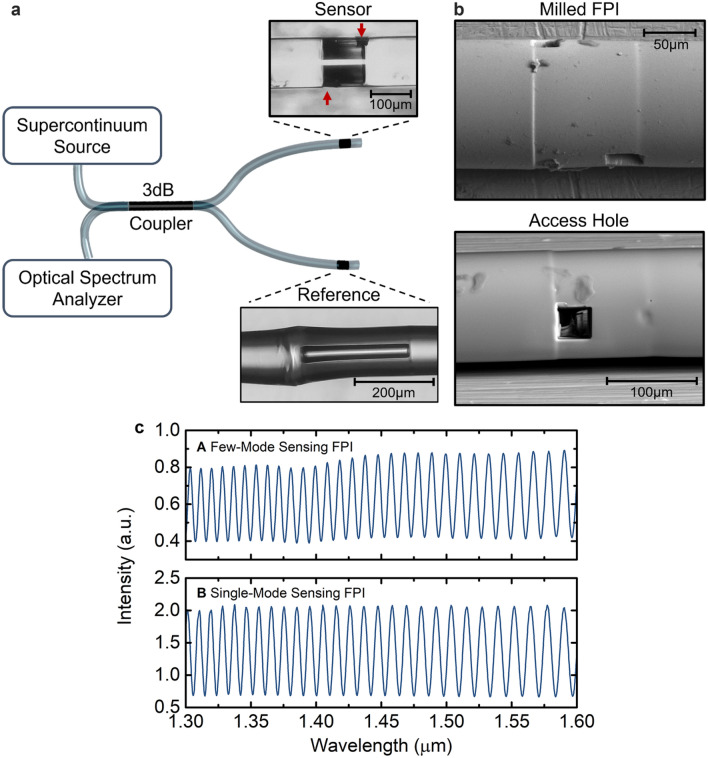
Fibre-optic refractometer based on the Vernier configuration. (**a**) Scheme of the Vernier effect in a parallel configuration. The sensor and reference interferometers are Fabry–Perot interferometers (FPIs) made from a hollow capillary tube. Two access holes for aqueous solutions are milled in the sensing FPI by means of a focused ion beam, whose details are provided in “Methods”. (**b**) Scanning electron microscope image of an example of a milled FPI and of an access hole. (**c**) Experimental spectra, before milling, of the few-mode sensing FPI in air (A) and of the single-mode sensing FPI in air (B).

In the experimental setup (Fig. 1a), the Vernier effect is generated in a parallel configuration, by means of a 3 dB fibre coupler^4^. It can be seen as a specific Michelson interferometer, however since the connecting parts are approximately invariant this approach for analysis of the parallel Vernier structure has not been used in literature^3,4,9,11–14^. The structure is illuminated with a supercontinuum source, and the reflected spectral response is measured with an optical spectrum analyser. The sensing and reference interferometers are FPIs, which can be fabricated using different methods^15–18^. In this work, the FPIs consist of a hollow capillary tube spliced between standard telecommunication single-mode fibres (further details in “Methods”). Two access holes milled near the splice regions of the sensing FPI by means of a focused ion beam enable the cavity to be filled with an aqueous solution (see “Methods”). An example image of a final milled FPI and of an access hole is depicted in Fig. 1b. The length of the sensing FPI is approximately 105 μm. The sensor was fabricated to excite few higher order modes in the sensing region by the introduction of a small offset in the input fibre (further details in “Methods”).

The spectrum of the few-mode sensing FPI in air, before milling the access holes is shown in Fig. 1c. A single-mode FPI structure, similar to the few-mode sensing FPI, was also fabricated for comparison purposes (see “Methods”), whose spectrum in air, before milling, is also depicted in Fig. 1c. There is a slight low-frequency modulation in the few-mode sensing FPI spectrum, which increases with longer wavelengths. However, the output spectrum of a single-mode sensing FPI is not modulated by such a low-frequency component as in the case of a few-mode sensing FPI.

**Figure 2:**
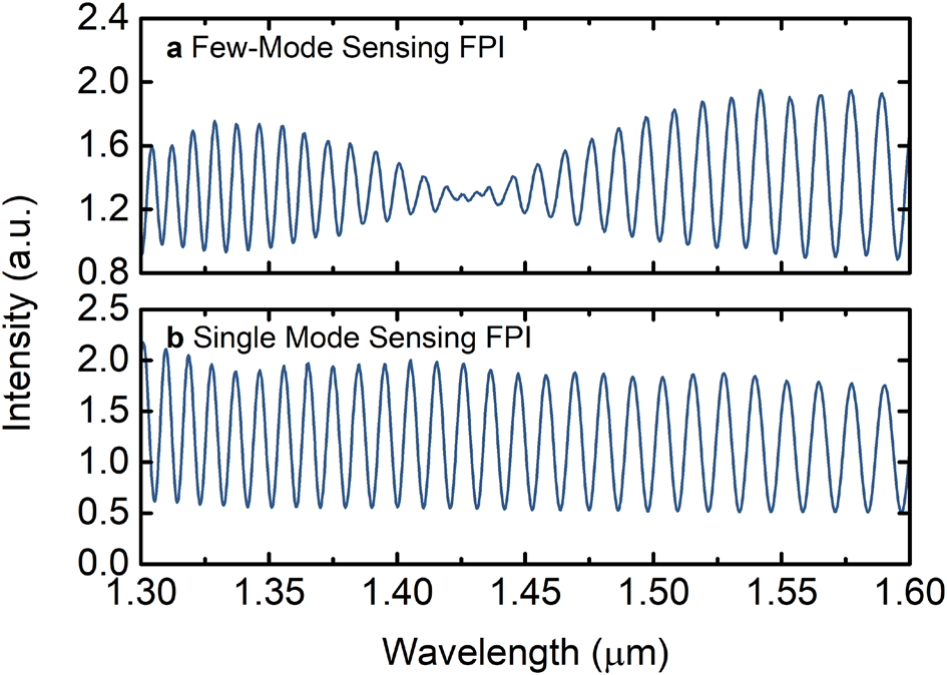
Intensity spectra after milling the access holes. (**a**) Few-mode sensing FPI. (**b**) Single-mode sensing FPI. The single-mode sensing FPI does not present a noticeable low-frequency modulation, while the few-mode sensing FPI shows a more predominant low-frequency modulation than before milling, with a node at around 1425 nm.

After milling the access holes, the visibility of the interference fringes of the output spectrum of the single-mode sensing FPI, represented in Fig. 2b, is approximately the same. Yet the output spectrum of the few-mode sensing FPI, presented in Fig. 2a, shows a more predominant low-frequency modulation than before milling (see Fig. 1c), with a node at around 1425 nm. Note that all the spectra correspond to FPIs in air. The existence of a node in the few-mode sensing FPI in the middle of the wavelength range monitored is inconvenient for generating the optical harmonic Vernier effect. This would result in a Vernier spectrum deformed at that wavelength region, which is problematic for tracing the upper Vernier envelope (or internal envelopes).

**Figure 3:**
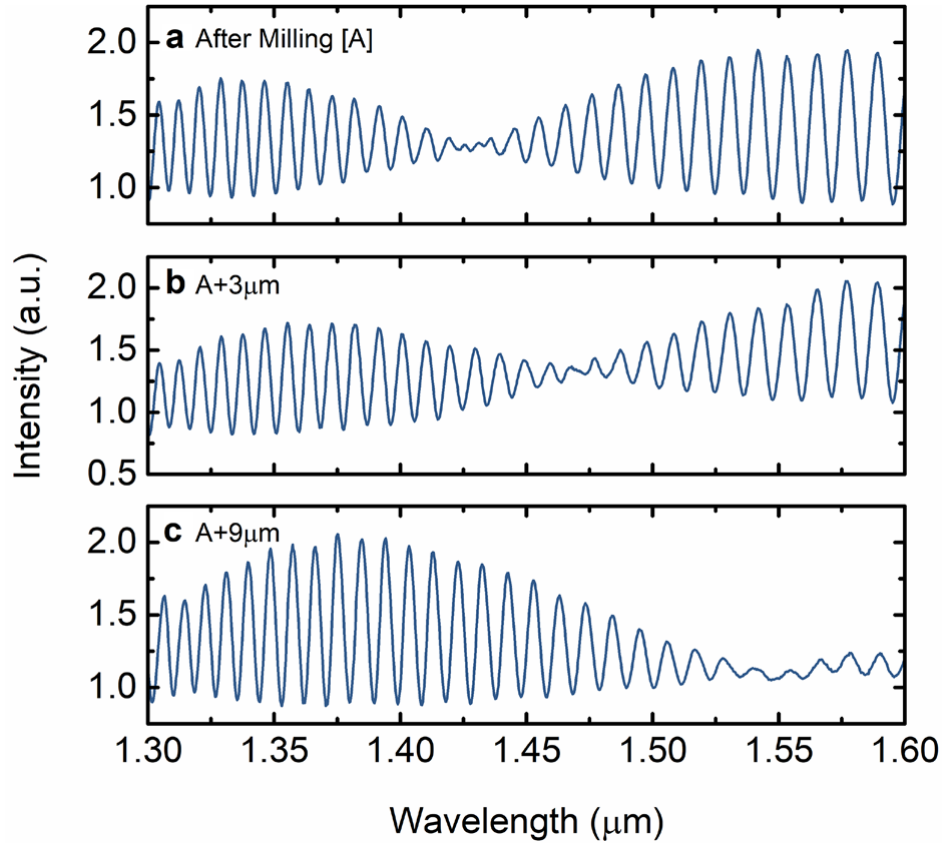
Intensity spectra of the few-mode sensing FPI after additional milling of the access holes. (**a**) Initial output spectrum [A] as in Fig. 2a. (**b**) Output spectrum after additionally milling 3 µm from the initial case [A]. (**c**) Output spectrum after additionally milling 9 µm from the initial case [A].

As an advantage, the milling process to open the access holes can be used to shift the low-frequency modulation of the output spectrum. By performing additional milling of the access holes by a few microns, the phase of the low-frequency modulation is slightly changed due to a slight variation of the effective refractive indices of the modes propagating in the FPI. As visible in Fig. 3, the position of the node is shifted towards longer wavelengths if an additional 3 µm are milled. After milling 9 µm from the initial case, the node of the low-frequency modulation shifted almost towards the end of the measured wavelength range, as shown in Fig. 3c.

**Figure 4:**
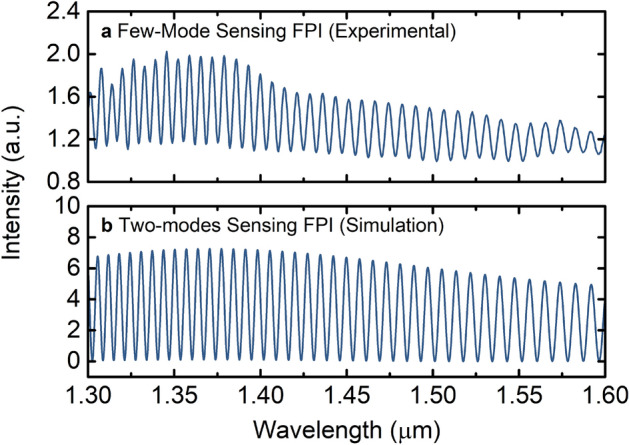
Comparison between the simulated two-mode sensing FPI and the experimental few-mode sensing FPI. (**a**) Experimental spectrum of the water-filled few-mode sensing FPI. (**b**) Simulated spectrum considering a two-modes sensing FPI.

The few-mode sensing FPI was then filled with deionized water, whose spectrum is shown in Fig. 4a. Instead of a clean sinusoidal behaviour characteristic of a single-mode FPI with low mirror reflectivities^19^, the measured signal is modulated by a non-uniform envelope. The envelope indicates the presence of other modes in the water-filled cavity, since it results from the interference between them. From the measured spectrum, the free spectral range (FSR) of the large envelope modulation is estimated to be around 450 nm. The effective refractive index difference between the fundamental mode (mode #1) and the main higher order mode (mode #2) that produce the envelope is in the order of 1.94 × 10^–2^ RIU (via Eq. 1 in “Methods”). Although this main higher order mode is expected to carry more energy than other higher order modes, the structure still presents other higher order modes that contribute to the FPI response, which is why the envelope is non-uniform. Such a structure is very challenging to simulate as it contains multiple variables and degrees of freedom. Therefore, as a first approach, let us consider a sensing FPI with only two modes. The simulated effective refractive index for the fundamental mode, LP_01_, of the fabricated sensing FPI is around 1.3150 RIU. Compared with the fundamental mode, the higher order mode, LP_012_, with an effective refractive index around 1.2954 RIU provides a similar refractive index difference as the one estimated previously from the experimental spectrum. The modelled spectrum for an FPI composed of these two modes, shown in Fig. 4b, has a main beating modulation very similar to the experimental spectrum (for further details on the simulations, see “Methods”). Other higher order modes do not give this high level of similarity with the experimental result. It is important to note that the higher order mode was derived solely from the experimental spectrum as the mode that fits best the experimental result.

### Proof-of-concept

The reference FPI was fabricated to be in tune with the fundamental mode of the sensing FPI, while generating the first optical harmonic of the Vernier effect^9^. Apart from having a twice higher magnification factor, the first harmonic provides traceable internal envelopes, whose intersections are easier to monitor than the traditional Vernier envelope^9^ (see “Methods”).

**Figure 5:**
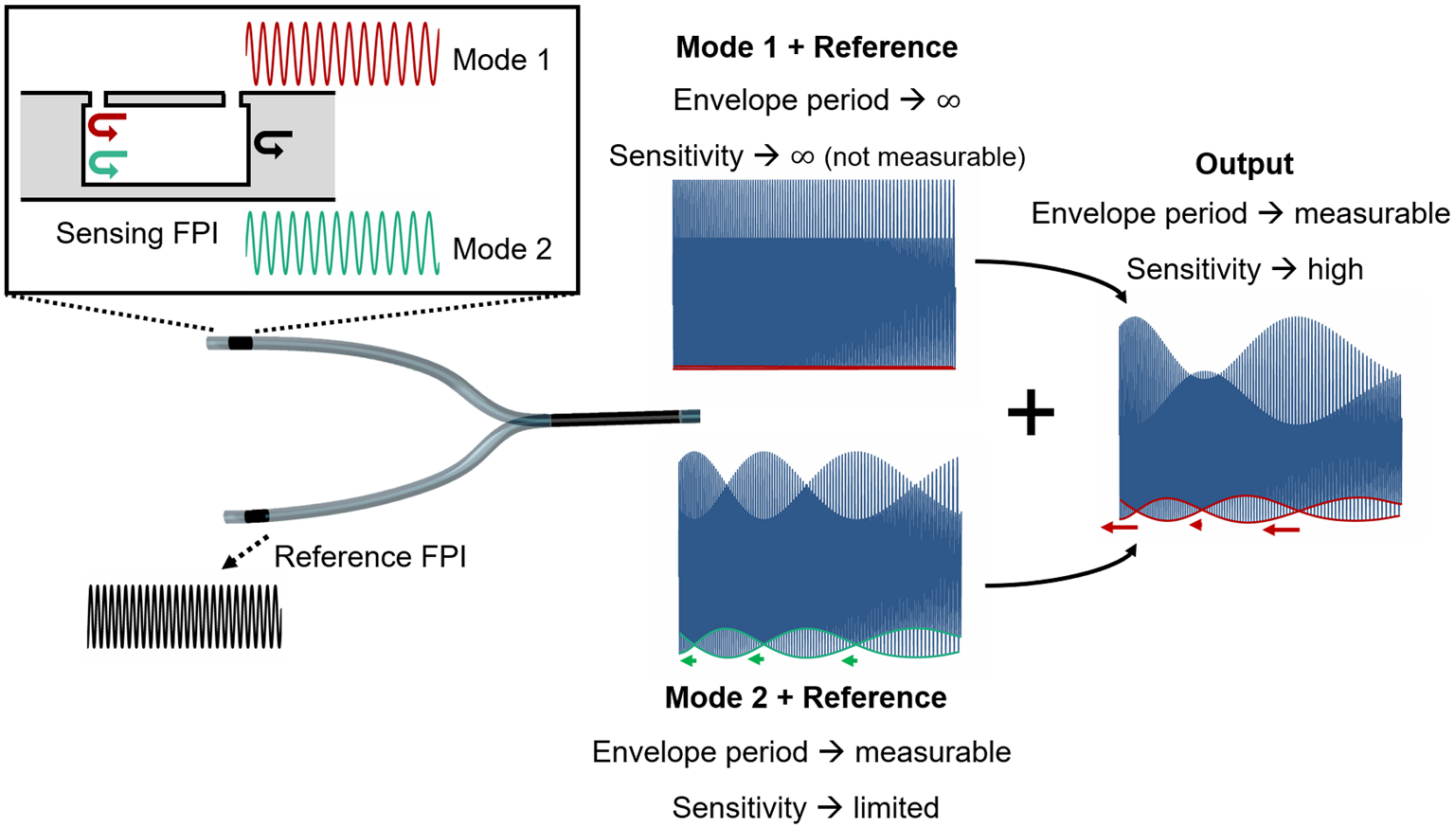
Schematic of the working principle considering two modes as an approximation. The signal produced by the two modes of the sensing FPI interfere with the reference FPI signal, producing the optical Vernier effect. The combined response at the output presents a complex modulation with envelope intersection points that achieve higher shift than in the normal case of optical Vernier effect, as shown qualitatively by the arrows. The Vernier spectra are represented in the range from 1.0 to 1.6 µm and are purely for explanation purposes, their visibilities were adjusted to be visually more perceptive and do not reflect the real case.

A simplified diagram of the working principle is depicted in Fig. 5. As a first approach, let us consider two modes in the sensing interferometer, represented in the inset of Fig. 5. The fundamental mode (mode 1) is in tune with the reference, as mentioned before, producing a signal modulated by an envelope with infinite period. In such case, the sensitivity is theoretically infinite, but one has no way of measuring it. As for a higher order mode (mode 2), it is slightly out of tune with the reference, producing a signal modulated by a Vernier envelope with a measurable period. As explained in the introduction, the sensitivity is then limited by the maximum size of the envelope that can be measured. A qualitative measurement of the envelope intersection shift for a varying measurand is represented in Fig. 5 by the arrows. The output of the structure is given by the overlap of these two cases, resulting in a more complex Vernier spectrum. In this situation, the envelope period is still measurable, presenting envelope intersections. However, some intersections show low sensitivity (short arrow length) while others present enhanced sensitivity (longer arrow length) comparing with the normal Vernier effect case. This effect is mainly the result of the relative movement between the envelopes in the complex spectrum, which is rather elaborate to analyse mathematically. Nevertheless, the enhanced sensitivity can be observed via simulations. From now on we are only interested in the region given by the envelope intersection that provide an enhanced wavelength shift.

**Figure 6:**
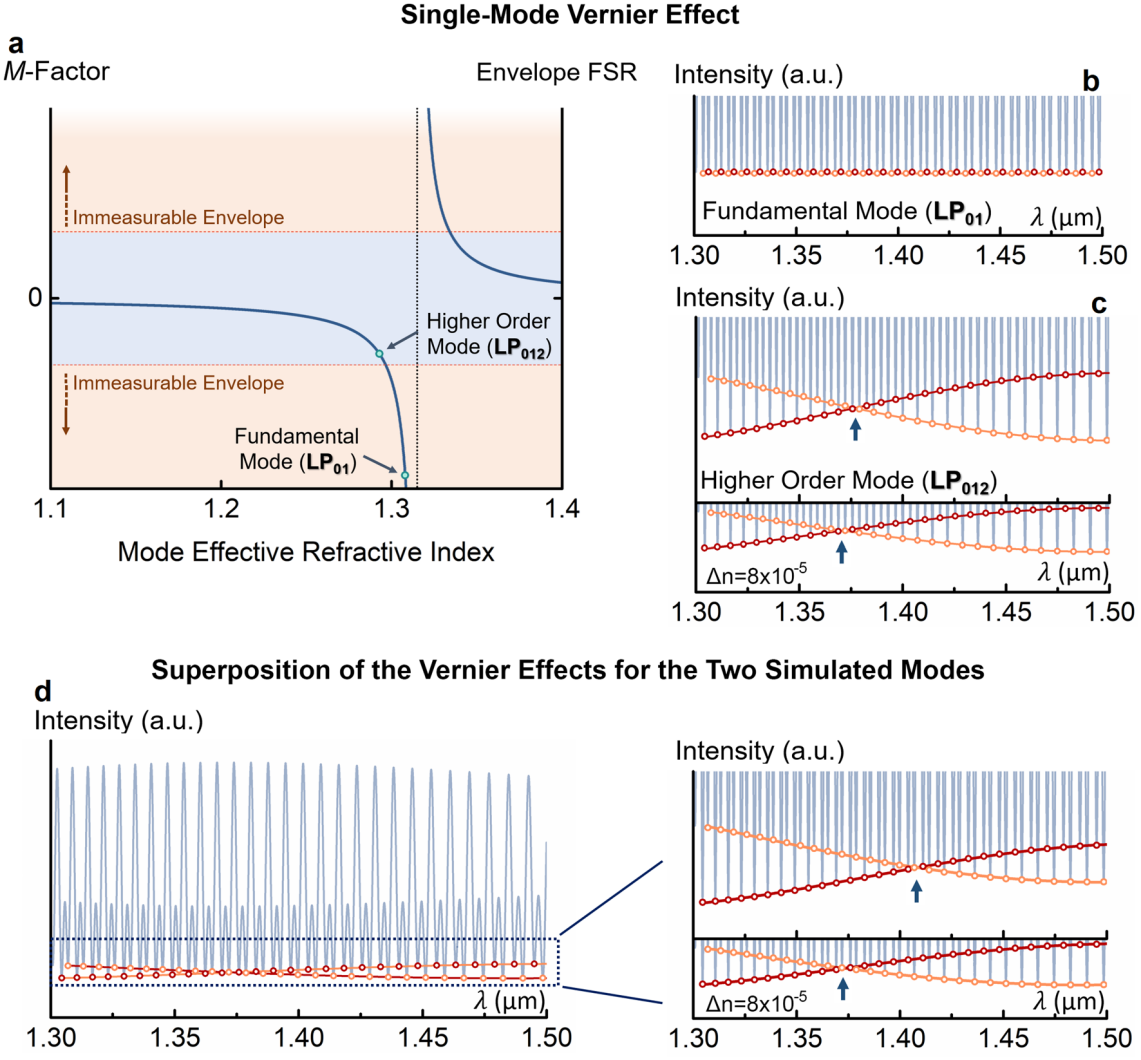
Comparison between the Vernier effect with a single mode and a two-mode sensing interferometer. (**a**) Magnification factor and envelope free spectral range (FSR) for a single mode sensing interferometer as a function of the mode-effective refractive index. (**b**) Simulated Vernier spectrum for a sensing interferometer with the fundamental mode (LP_01_). The Vernier spectrum has a high magnification factor, but an envelope too large to be measured. (**c**) Simulated Vernier spectrum for a sensing interferometer with the higher order mode (LP_012_). The Vernier envelope is measurable but has a lower magnification factor (lower wavelength shift). (**d**) Simulated Vernier spectrum for a two-mode sensing interferometer. The Vernier envelope is measurable, yet the magnification factor is still high (larger wavelength shift than the single mode case). These two last cases involved the same refractive index variation of the analyte by 8 × 10^–5^ RIU.

The air-filled reference FPI was produced with a length close to 276.2 μm (details in “Methods”). The optical path length of the reference FPI should closely match twice the optical path length of the fundamental mode for the water-filled sensing FPI, but still be slightly longer. In this situation, the magnification factor for all the modes of the sensing FPI is negative (Fig. 6a) (via Eq. 9 in “Methods”). A negative magnification factor simply means a wavelength shift of the Vernier envelope in the opposite direction compared to the normal sensing FPI.

For this specific dimensioning of the FPIs in the Vernier structure, let us interpret now the outcome of a standard situation where the sensing FPI is a single-mode one. If the sensing FPI only presents the fundamental mode, which is in tune with the reference FPI, the generated Vernier envelope would present an extremely large FSR and a high magnification factor. In practice however, it would be impossible to measure the Vernier envelope shift within the limited spectral range available, as seen in Fig. 6b. On the other hand, if the sensing FPI only presents the higher order mode LP_012_, the Vernier effect is less tuned, resulting in a smaller period Vernier envelope. In this case, the Vernier envelope is now measurable, but it is accompanied by a smaller magnification factor and, therefore, by a smaller wavelength shift, as demonstrated in Fig. 6c. Hence, in a standard single-mode situation, the maximum magnification factor provided by the Vernier effect is limited by the largest Vernier envelope measurable. However, if the sensing FPI presents both modes simultaneously, the superposition of both responses results in a measurable Vernier envelope but with a higher magnification factor (larger wavelength shift), as observed in Fig. 6d. This result, simulated for a two-mode sensing FPI, is expected to still be applicable to the fabricated sensing FPI, which may present some additional modes (few mode case).

## Experimental results

**Figure 7:**
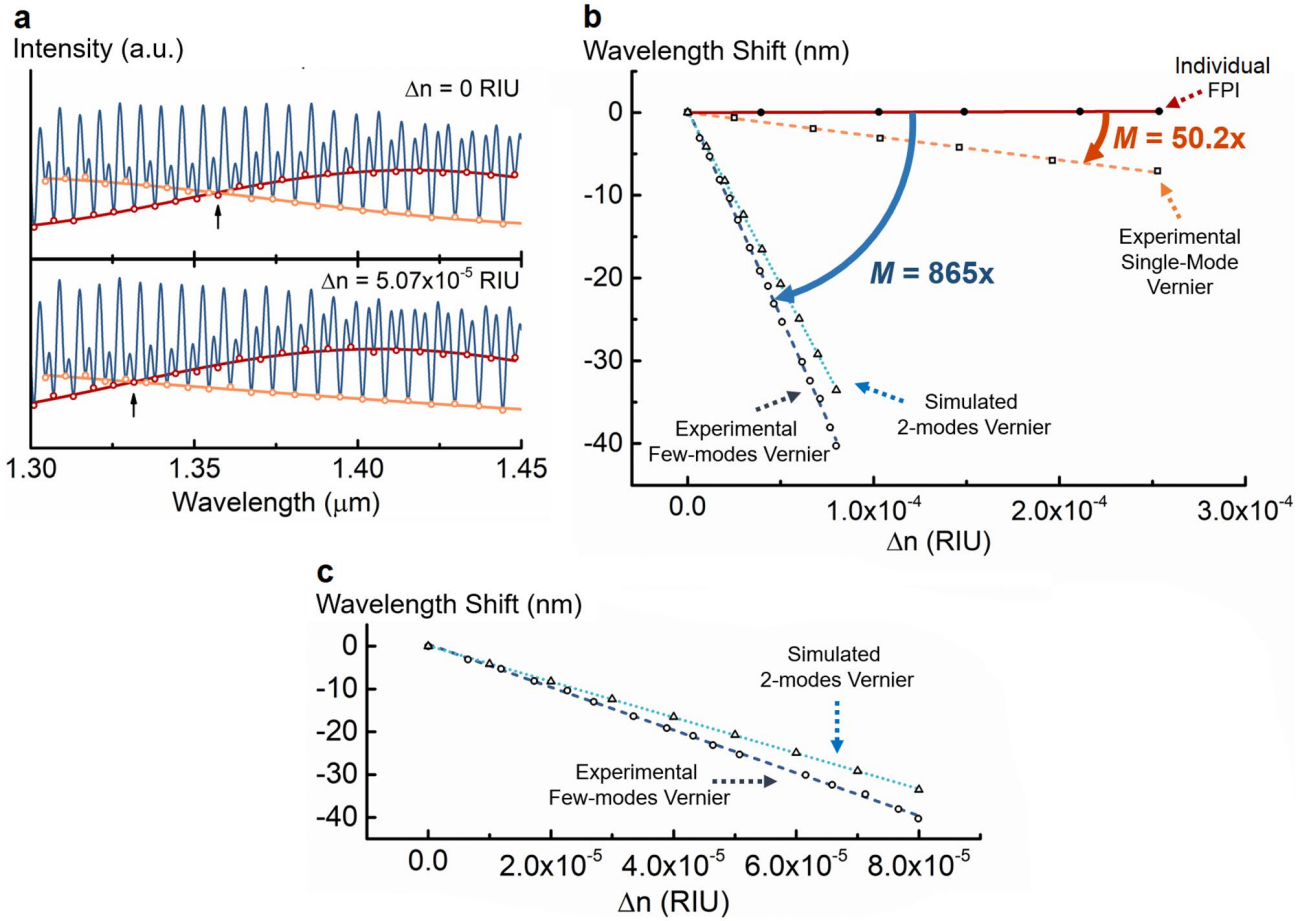
Characterization results. (**a**) Experimental Vernier spectra for a few-mode water-filled sensing interferometer at different refractive indices. The internal Vernier envelope intersection, marked with an arrow, is traced and monitored during the characterization. (**b**) Wavelength shift as a function of water refractive index variations for different configurations: individual sensing Fabry–Perot interferometer (FPI), experimental Vernier effect for a single mode sensing FPI, simulated Vernier effect for a two-mode sensing FPI, and experimental Vernier effect for a few-mode sensing FPI. The magnification factor (*M*) achieved by the Vernier effect with a few-mode sensing FPI is an order of magnitude higher than the Vernier effect with a single mode sensing FPI. (**c**) Magnification of the wavelength shift for the simulated Vernier effect with a two-mode sensing FPI compared with the experimental Vernier effect with a few-mode sensing FPI.

The experimental Vernier spectrum for the fabricated few-mode sensing FPI filled with water is shown in Fig. 7a. The performance of the structure was evaluated by applying it as a refractometer. The sensor was characterized for refractive index variations in a very narrow range around the refractive index of water. This was achieved by slightly changing, in steps, the water temperature, which, through the thermo-optic effect, changes the refractive index of water (see “Methods”). In total, the refractive index of water changed by 7.989 × 10^–5^ RIU. In each step, the output spectrum was recorded and the position of the intersection between the internal Vernier envelopes was monitored as a function of the refractive index variation. Figure 7a also presents an example of the experimental spectral shift for a refractive index variation of 5.07 × 10^–5^ RIU. The wavelength shift of the Vernier envelope as a function of water refractive index variations is depicted in Fig. 7b. We observe a high refractive index sensitivity of − 500,699 nm/RIU. For comparison purposes, a similar sensing structure was fabricated to generate the first harmonic of the Vernier effect, but with the single-mode sensing FPI previously analysed (details in “Methods”). Its response is also displayed in Fig. 7b, together with the individual response of the sensing FPI and the simulated response for a simplified Vernier structure with only a two-mode sensing FPI (details in “Methods”). The sensitivity of the sensing FPI alone is 568 nm/RIU, which was improved to − 28,496 nm/RIU by adding the first harmonic of the Vernier effect with a single-mode sensing FPI. Such improvement corresponds to a magnification factor of around 50.2. On the other hand, the magnification factor of the proposed Vernier structure with a few-mode sensing FPI is higher than 850, which is an order of magnitude higher than the magnification factor obtained with the single-mode Vernier structure. The sensitivity of the simplified simulated Vernier structure with only a two-mode sensing FPI is around − 418,387 nm/RIU. The result for the experimental structure with few modes is in the same order of magnitude as the simulated structure considering only two modes. It is important to note that the few-mode sensing FPI still presents modes other than the two considered for simulations, which contribute to a slight further increase of the magnification factor. Additionally, the thermal expansion of the FPI was not considered, as it only introduces an error of about 1.3% (see “Methods”).

The response of the structure is limited by the resolution of the detection system, corresponding to a refractive index resolution of 5 × 10^–7^ RIU. In theory, a resolution of 2 × 10^–9^ RIU could be achieved by using a detection system with a resolution of 1 pm, which nowadays is commercially available. Monitoring the time dependent phase of the Vernier envelope would be a different way to characterize the structure. However, such approach is, at this point, not feasible due to the limitations of the available interrogation system.

## Discussion

The demonstrated method that combines an extreme optical Vernier effect with a few-mode sensing interferometer proved to be capable of overcoming the limitations of the standard optical Vernier effect techniques. The implementation of a few-mode fibre refractometer with such method registered a record magnification factor of 865, also reaching a record value for refractive index sensitivity for this kind of interferometric fibre structure. In fact, for a single-mode Vernier structure to achieve an extreme magnification factor of 865, it would correspond to an immeasurable Vernier envelope FSR longer than 6400 nm. Therefore, the proposed method allows us to achieve such huge magnification factors whilst maintaining a measurable envelope.

From the obtained results for refractive index sensing, it would be very attractive to apply a similar structure for high resolution gas sensing and biosensing, namely in extreme environments, where it is necessary to detect very small concentrations. The proposed concept can also be adapted to other sensing interferometers (Mach–Zehnder interferometers, Michelson interferometers, hybrid interferometric structures, among others), to obtain giant sensitivities to other physical and chemical parameters. Therefore, the proposed method opens new horizons for the development of a new generation of sensors with extreme sensitivities and resolutions for demanding state-of-the-art applications.

## Methods

### Fabrication of the Fabry–Perot interferometers

As illustrated in Fig. 1a, the Fabry–Perot interferometers are based on a section of a hollow capillary tube spliced between two pieces of a single mode telecommunication fibre (SMF-28). The hollow capillary tube used for the sensing Fabry–Perot interferometer has an outer diameter of 125 μm and an internal diameter of 80 μm. For the reference Fabry–Perot interferometer, the hollow capillary tube used has an internal diameter of 60 µm. Both hollow capillary tubes were fabricated at Leibniz-IPHT. Initially, the cleaved end of a single mode fibre and a hollow capillary tube were spliced together with a splicing machine (Fitel S177). The electric arc was centred mainly in the single mode fibre by means of the manual mode of the fusion splicer; thereby we avoided the collapse of the capillary tube^9^. Two electric arcs were applied with an arc power of 30 arbitrary units and arc duration of 400 ms. Following this procedure, the hollow capillary tube was cleaved for the desired length with the assistance of a magnification lens. Finally, the cleaved end of the input single mode fibre was spliced to the cleaved end of the capillary tube by the same procedures as described before. For the few-mode sensing FPI, an extra step is required during the initial splice with the input single mode fibre. The splice was performed with a slight offset of 5 arbitrary units and with the same arc power and duration (30 arbitrary units and 400 ms), followed by two compressions of 15 arbitrary units, each of them accompanied by a cleaning arc. Due to the offset, higher order modes are excited in the hollow capillary tube section. Further details on the reproducibility can be found below in “Methods”.

### Milling of access holes

Two access holes for liquid analytes were milled in the sensing Fabry–Perot interferometer with a Tescan (Lyra XMU) focused ion-beam scanning electron microscope (FIB-SEM). Before milling, the sample was placed on an aluminium holder and fixed with carbon glue (DOTITE XC-12, Fujikura Kasei Co., Ltd. Tokyo, Japan). To use the focused ion beam in optical fibres, it is necessary to suppress the surface charging effects by depositing a thin layer of a conductive material. Unwanted milled regions and inaccurate milling geometries can arise from ion beam drifting effects due to charge accumulation in the sample. For this reason, the few-mode sensing interferometer structure was carbon-coated by means of a LEICA EM ACE600. Since the milling of the access holes is a long-time process, it is useful to have a conductive film of a material with a low milling rate. In general, carbon is more stable and presents a lower milling rate compared with other conductive films typically employed for the same purpose, such as platinum or gold. The sample was placed at a working distance of 50 mm, with stage rotation, and no stage tilt. The coating was performed with a chamber pressure of 9 × 10^–6^ bar. A total of 6.4 nm of carbon was deposited on the sample. The first access hole was milled near a splice region, with a sample tilt of − 20°. A section of 25 μm × 25 μm with a depth of 25 μm was initially milled with an ion current of 7 nA. Then, the access hole was further expanded with the same ion current by a milling strategy normally applied for polishing. The final dimension of the first access hole was 32 μm x 31 μm. The second access hole was similarly milled at the other splice region. The sample was rotated 180º and tilted by − 15°. A section of 20 μm x 10 μm with a depth of 25 μm was initially milled with an ion current of 7.1 nA. At this point it was necessary to deposit a new carbon coating due to the removal of much of the previous coating during the milling process. This time, the carbon coating was performed in the direction of the milling region, tilting the sample by 10° and applying no stage rotation. A thickness of 9.56 nm of carbon was deposited at a chamber pressure of 2.5 × 10^–5^ bar. Following this, the second access hole was further expanded with an ion current of 6 nA by a milling strategy normally applied for polishing. The final dimension of the second access hole was 25 μm x 24 μm. No disturbing effects of volume charging related with drifting effects were observed during the milling processes.

The access holes of the single-mode sensing interferometer were fabricated with the same procedure. In this case, the first access hole was milled with an ion current of 6.9 nA and a sample tilt of − 15°. The final dimension of the first access hole was 26 μm x 21 μm. The second access hole was milled with an ion current of 5.9 nA, the same tilt and a sample rotation of 180°. The final dimension of the second access hole was 20 μm x 18 μm.

In all the cases, the milled interferometers were kept fixed to the sample holder with carbon glue. This helps to maintain the stability of the structure and avoids its movement during its usage, which can induce a fracture at the milled region.

### Calculation of effective refractive index difference through experimental intensity spectrum

In a two-mode Fabry–Perot interferometer, the effective refractive index difference $\left( {\Delta n} \right)$ between the fundamental mode and a higher order mode that produce an envelope modulation in the reflection spectrum can be calculated through^20^:

1 $$ \Delta n = \frac{{\lambda_{1} \lambda_{2} }}{2L \times FSR}, $$

where $\lambda_{1}$ and $\lambda_{2}$ are the wavelength positions of two consecutive maxima (or minima) of the envelope, *L* is the length of the sensing interferometer, and *FSR* is the free spectral range of the envelope modulation. For the few-mode sensing interferometer developed, which presents a length of 105 μm, the *FSR* is estimated to be around 450 nm. Half a period of the envelope modulation is located between 1375 and 1600 nm. Hence, the wavelength values in Eq. 1, corresponding to the position of two consecutive envelope minima, are assumed as 1150 nm and 1600 nm. With this, a refractive index difference of around 1.94 × 10^–2^ RIU is obtained through Eq. 1.

### Mode simulation in the sensing Fabry–Perot interferometer

**Figure 8:**
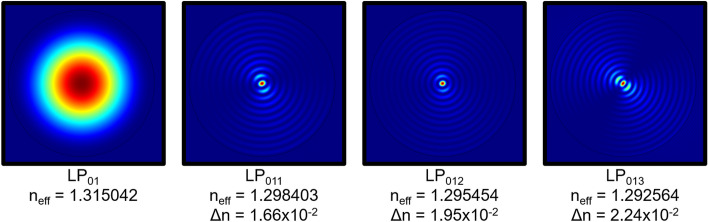
Simulated mode profiles. Mode profile of the fundamental mode and the three higher order modes with an effective refractive index difference close to the value calculated through the experimental data (1.94 × 10^–2^ RIU).

Mode analysis using COMSOL Multiphysics was performed to calculate the effective refractive index of modes propagating in the water-filled sensing Fabry–Perot interferometer. The simulated cross-section consists of a capillary tube made of silica (refractive index of 1.444 at 1550 nm^21^) with an inner diameter of 80 μm and an outer diameter of 125 μm. The inner region of the capillary tube and the external environment correspond to water at 22.72 °C (refractive index of 1.315107 at 1550 nm, calculated through Eq. 11 in “Methods”). The simulated fundamental mode (LP_01_) has an effective refractive index of 1.315042. Its mode profile is depicted in Fig. 8. The simulated higher order modes LP_011_, LP_012_ and LP_013_, also shown in Fig. 8 for comparison purposes, have an effective refractive index difference, in relation to the fundamental mode, close to the value calculated through the experimental data (1.94 × 10^–2^ RIU, see Method section before).

**Figure 9:**
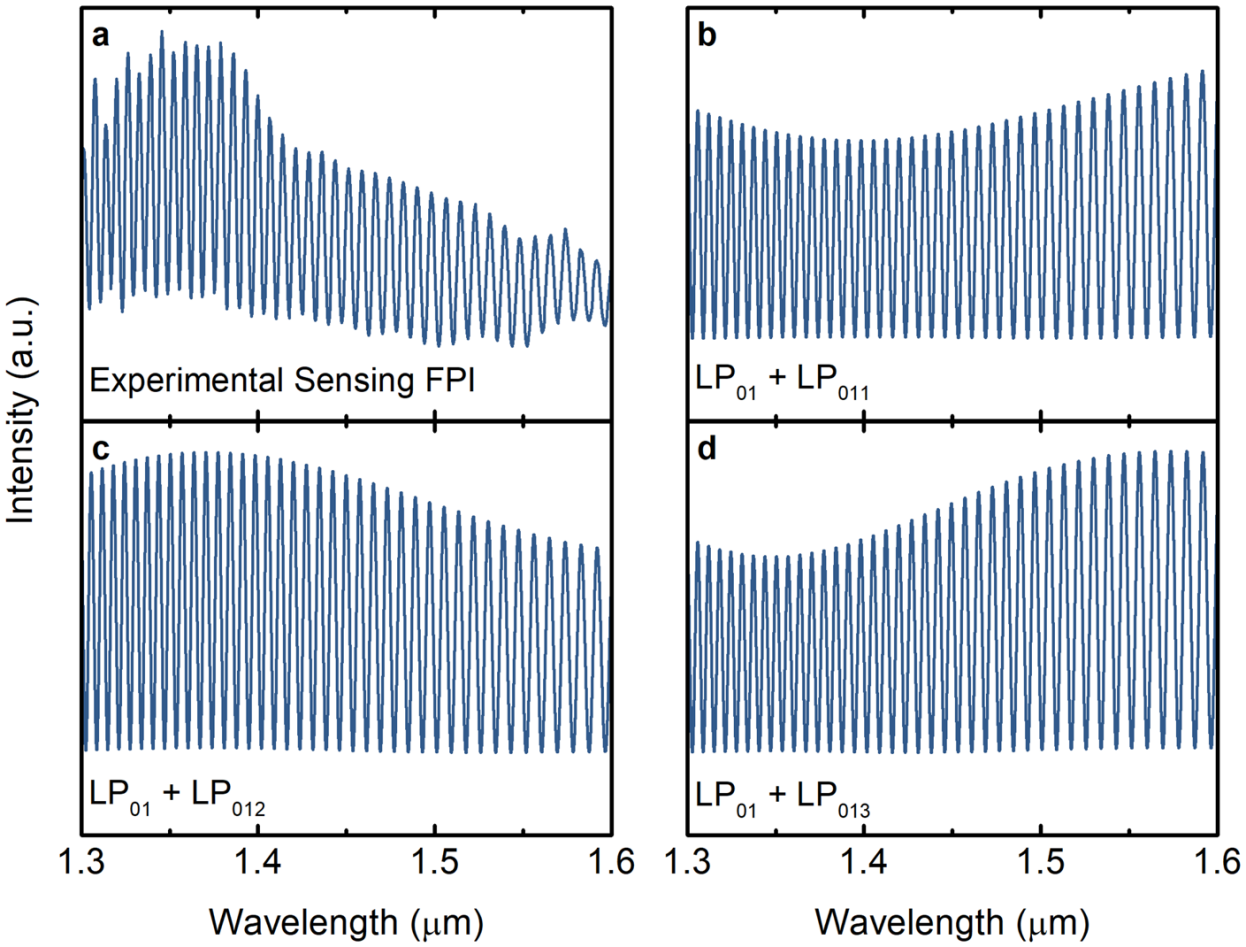
Intensity spectrum of the sensing interferometer. (**a**) Experimental intensity spectrum of the few-mode sensing interferometer. Simulated intensity spectra for a two-mode sensing interferometer with: (**b**) fundamental mode LP_01_ and higher order mode LP_011_; (**c**) fundamental mode LP_01_ and higher order mode LP_012_; (**d**) fundamental mode LP_01_ and higher order mode LP_013_.

The mode LP_012_, which presents the closest effective refractive index difference, was taken as the second mode for the simulated two-mode sensing interferometer. Figure 9 shows the experimental few-mode sensing Fabry–Perot interferometer spectrum and the simulated spectra for two-mode sensing Fabry–Perot interferometers consisting of the fundamental mode and one of the three higher order modes as determined before. The simulated normalized intensity spectrum $\left( {I_{out} \left( \lambda \right)} \right)$ for a two-mode Fabry–Perot interferometer is described by

2 $$ I_{out} \left( \lambda \right) = \left| {\frac{{E_{out} \left( \lambda \right)}}{{E_{in} \left( \lambda \right)}}} \right|^{2} = \frac{{E_{out} \left( \lambda \right) \cdot E_{out}^{*} \left( \lambda \right)}}{{E_{in}^{2} \left( \lambda \right)}}, $$

where $E_{out}^{*} \left( \lambda \right)$ is the complex conjugate of $E_{out} \left( \lambda \right)$. $E_{out} \left( \lambda \right)$ and is given by

3 $$ E_{out} \left( \lambda \right) = E_{in} \left( \lambda \right)\left\{ {A + f_{1} Bexp\left[ { - i\left( {\frac{4\pi }{\lambda }n_{LP0,1} L - \pi } \right)} \right] + f_{2} Bexp\left[ { - i\left( {\frac{4\pi }{\lambda }n_{LP0,m} L - \pi } \right)} \right]} \right\}, $$

where *L* is the length of the Fabry–Perot interferometer, $n_{LP01}$ and $n_{LP0m}$ are the effective refractive indices of the fundamental mode considered and the higher order mode *m*, respectively; $\lambda$ is the wavelength, and $E_{in} \left( \lambda \right)$ is the input electric field. The coefficient *A* is given by

4 $$ A = \sqrt {R_{1} } , $$

with *R*_*1*_ being the intensity reflection coefficient of the first interface of the Fabry–Perot interferometer. The coefficients *B* is given by

5 $$ B = \left( {1 - R_{1} } \right)\sqrt {R_{2} } , $$

where *R*_*2*_ is the intensity reflection coefficient of the second interface of the Fabry–Perot interferometer. The factors *f*_1_ and *f*_2_ correspond to the percentage of power distributed to the fundamental mode and to the higher order mode, respectively. To approach a real situation, where the fundamental mode carries a lot more power than the higher order mode, in this simulation we considered *f*_1_ as 85% and *f*_2_ as 15%. The length of the Fabry–Perot interferometer was considered as 105 μm. To also address some losses due to the slight offset of the input fibre and losses due to surface imperfections and mode mismatch, the coefficient A was reduced by 20%.

Comparing the intensity spectra of Fig. 9, one can conclude that the two-mode sensing Fabry–Perot interferometer with the higher order mode LP_012_ fits the experimental result best.

### Fitting internal envelopes in the optical harmonic Vernier effect

The optical harmonic Vernier effect enables the possibility of tracing internal envelopes, providing an intersection point that is easier to monitor in comparison to the typical upper or lower Vernier envelope. In the parallel configuration, as used in this work, the number of internal envelopes $\left( {N_{internal\;envelopes} } \right)$ is related to the harmonic order as

6 $$ N_{internal\;envelopes} = i + 1, $$

with *i* being the harmonic order. Therefore, for the first harmonic of the Vernier effect, one can trace two internal envelopes. The internal envelopes are formed by grouping peaks (either maxima or minima) in the harmonic Vernier spectrum. For the first harmonic of the Vernier effect, one can form two groups of peaks. In this work, we choose to trace the minima. Hence, we grouped every 2*m* peak of the intensity spectrum in a linear scale, with *m* being the number of the peak, and fitted them with a cosine function. The same was performed for the other group of peaks, consisting of every 2* m* + 1 peak. At the end, the intersection between the two internal envelopes was monitored for sensing applications.

### Calculation of the reference interferometer length

The sensing Fabry–Perot interferometer has a length of 105 μm. To generate the first harmonic of the Vernier effect to be in tune with the fundamental mode of the sensing Fabry–Perot interferometer $\left( {n_{eff} = 1.315042} \right)$, the optical path length of the reference interferometer should match closely twice the optical path length (OPL) of the sensing interferometer, as described by

7 $$ OPL_{reference} \approx 2 \times OPL_{LP0,1} \leftrightarrow n_{reference} L_{reference} = 2 \times n_{effsensing} \times L_{sensing} = 276.159\;\upmu {\text{m}}. $$

In fact, to provide a huge magnification factor, the OPL of the reference interferometer should be slightly detuned from twice the OPL of the sensing interferometer. Therefore, the OPL of the reference interferometer is expressed as

8 $$ OPL_{reference} = 2 \times OPL_{LP0,1} - 2\Delta , $$

where $\Delta$ is the detuning parameter. Twice the detuning parameter $\Delta$ corresponds to the optical path difference between the actual reference interferometer and the closer situation of a perfect harmonic case (where $OPL_{2} = \left( {i + 1} \right)OPL_{1}$, *i* being the order of the harmonic. For the first harmonic, *i* = 1). In other terms, $\Delta = 2n_{sensing} L_{sensing} - n_{reference} L_{reference}$.

Since the reference interferometer is made of an air-filled cavity, the reference interferometer length is approximately half the optical path length, as the refractive index is about 1. The magnification factor for the first harmonic of the Vernier effect is then approximately given by^9^

9 $$ M^{1st\;Harmonic} = \frac{{2n_{sensing\;interf.} L_{sensing\;interf.} }}{\Delta }, $$

To obtain a negative magnification factor, the optical path length of the reference interferometer should be larger than the optical path length of the sensing interferometer, so that the detuning parameter ($\Delta$) in Eq. 9 becomes negative. Hence, the length of the reference interferometer was considered as 276.2 μm, slightly larger than 276.159 μm.

### Sensor characterization

The sensing FPI was immersed in a deionized water bath, where the water was heated up to 23.46 °C and then slowly decreased to 22.72 °C, while simultaneously the sensor spectrum was monitored. The water temperature was monitored by means of a thermocouple (Almemo 1020-2, with a thermoelement Type N) having a resolution of 0.01 °C, placed closely to the sensing FPI.

The refractive index of water at each temperature was calculated through the thermo-optic coefficient $\left( {{{dn} \mathord{\left/ {\vphantom {{dn} {dT}}} \right. \kern-\nulldelimiterspace} {dT}}} \right)$ at a wavelength of 1550 nm, which is given by^22^

10 $$ \frac{dn}{{dT}} = - 1.543 \times 10^{ - 7} T - 1.044 \times 10^{ - 4} . $$

Considering the refractive index of water at 20 °C as 1.3154, at a wavelength of 1550 nm^23^, Eq. 10 can be integrated, which yields an expression for the refractive index of water at a given temperature:

11 $$ n\left( T \right) = 1.3154 + 2.11886 \times 10^{ - 3} - 1.044 \times 10^{ - 4} T - 7.715 \times 10^{ - 8} T^{2} , $$

with *T* given in °C.

If the measured liquid needs to be changed, the transducer should be cleaned with isopropanol and dried before using it again.

### Effect of thermal expansion

The typical temperature sensitivity of a Fabry–Perot interferometer given by a hollow capillary tube, due to thermal expansion of the structure, is in the order of 0.83 pm/°C^24,25^. This sensitivity value is also magnified by the optical Vernier effect (865x), which means that the sensitivity of the Vernier envelope due to thermal expansion is around − 718 pm/°C (note that the magnification factor is negative, which results in a negative response of the Vernier envelope). Converting the experimental sensitivity to refractive index $\left( {S_{RI} = - 500699\;{\text{nm}}/{\text{RIU}}} \right)$ into the corresponding temperature sensitivity $\left( {S_{T} } \right)$, one obtains:

12 $$ S_{T} = S_{RI} \frac{\Delta RI}{{\Delta T}} = - 500699 \cdot \frac{{7.989 \times 10^{ - 5} }}{0.74} = - 54.055\;[nm/RIU], $$

where $\Delta T$ is the temperature variation used in the experiment (from 23.46 °C to 22.72 °C) and $\Delta RI$ is the correspondent refractive index variation due to the thermo-optic effect, as previously discussed.

The value of temperature sensitivity (− 54,055 pm/°C) corresponds actually to the sum between the thermo-optic effect and the thermal expansion. By subtracting the thermal expansion estimated before (− 718 pm/°C), the temperature sensitivity due only to the thermo-optic effect is − 53,377 pm/°C. Hence, the thermal expansion was negligible during the experiments, as it is only about 1.3% of the final value.

### Fabrication of the single-mode sensing Fabry–Perot interferometer

The single-mode sensing Fabry–Perot interferometer was fabricated using the same procedures as described in “Methods”. The length of the interferometer is approximately 101 μm. A reference Fabry–Perot interferometer was fabricated to generate the first harmonic of the Vernier effect. Using Eq. 7, the length of the reference interferometer should closely match 265.6 µm, but still be slightly larger. Hence, the fabricated reference Fabry–Perot interferometer has a length of around 269.5 µm.

### Simulation of Vernier effect with a two-mode sensing Fabry–Perot interferometer

In a parallel configuration using a 50/50 fibre coupler, the reflected intensity spectrum at the output is given by the overlap between the signals reflected from both interferometers in the fibre coupler. The output electric field, then, is the sum between the electric field reflected from the sensing interferometer, which for a two-mode Fabry–Perot interferometer is described by Eq. 3, and the electric field reflected from the reference interferometer. Subsequently, the output electric field is expressed as

13 $$ \begin{aligned} E_{out} \left( \lambda \right) & = \frac{{E_{in} \left( \lambda \right)}}{\sqrt 2 }\left\{ {A + f_{1} Bexp\left[ { - i\left( {\frac{4\pi }{\lambda }n_{LP0,1} L_{1} - \pi } \right)} \right] + f_{2} Bexp\left[ { - i\left( {\frac{4\pi }{\lambda }n_{LP0,12} L_{1} - \pi } \right)} \right]} \right\} \\ & \quad + \frac{{E_{in} \left( \lambda \right)}}{\sqrt 2 }\left\{ {C + Dexp\left[ { - i\left( {\frac{4\pi }{\lambda }n_{2} L_{2} - \pi } \right)} \right]} \right\}, \\ \end{aligned} $$

where *L*_1_ is the length of the two-mode sensing Fabry–Perot interferometer, $n_{LP0,1}$ and $n_{LP0,12}$ are the effective refractive indices of the considered fundamental mode and the higher order mode, respectively, in the two-mode sensing Fabry–Perot interferometer, *n*_2_ and *L*_2_ are the effective refractive index and the length of the reference interferometer, $\lambda$ is the wavelength, and $E_{in} \left( \lambda \right)$ is the input electric field. The coefficients *A*, *B*, $f_{1}$ and $f_{2}$ are the same as in the mode simulation section in “Methods”. The coefficient *C* is given by

14 $$ C = \sqrt {R_{1}^{ref} } , $$

with $R_{1}^{ref}$ being the intensity reflection coefficient of the first interface of the reference Fabry–Perot interferometer, which has an air cavity (the refractive index was considered as 1). The coefficient *D* is given by

15 $$ D = \left( {1 - R_{1}^{ref} } \right)\sqrt {R_{2}^{ref} } , $$

where $R_{2}^{ref}$ is the intensity reflection coefficient of the second interface of the reference Fabry–Perot interferometer. The output intensity spectrum is obtained by substituting Eq. 2 for Eq. 13.

The length of the sensing Fabry–Perot interferometer was considered as 105 µm. The effective refractive index of the modes LP_01_ ($n_{LP01}$) and LP_012_ ($n_{LP012}$) are 1.315042 and 1.295454, respectively, the same as simulated in the mode simulation section in “Methods”. To approach a real situation where the fundamental mode carries a lot more power than the higher order mode, in this simulation we considered $f_{1}$ as 85% and $f_{2}$ as 15%. To also address some losses due to the small offset of the input fibre, the coefficient *A* was reduced by 20%. In the same way, the coefficient *D* was reduced by 70% to adjust the intensity due to losses during the fine-tuning of the reference interferometer by compression. The refractive index of the reference Fabry–Perot interferometer was considered as 1 (air). The optical path length of the reference Fabry–Perot interferometer was chosen to closely match twice the optical path length for the fundamental mode of the sensing Fabry–Perot interferometer, but still be slightly longer. By this way, the first harmonic of the Vernier effect is generated. Hence, the length used for the reference interferometer is 2 × 1.315042 × 105 µm + 0.001 µm. If the sensing Fabry–Perot interferometer were single-mode, this would correspond to a situation where the generated Vernier envelope would be approximately infinite and have an infinite magnification factor. The final simulated spectrum for the first harmonic of the Vernier effect with a two-mode sensing Fabry–Perot interferometer is depicted in Fig. 6b. To simulate the response of this structure to refractive index variations, the refractive index was successively reduced by steps of 10^–5^ RIU until a maximum change by 8 × 10^–5^ RIU. For simplification, we assumed that the effective refractive indices of both modes in the sensing Fabry–Perot interferometer are reduced identically by 10^–5^ RIU in each step. The internal envelope intersection is monitored in each step, producing the wavelength shift depicted in Fig. 7b,c.

**Figure 10:**
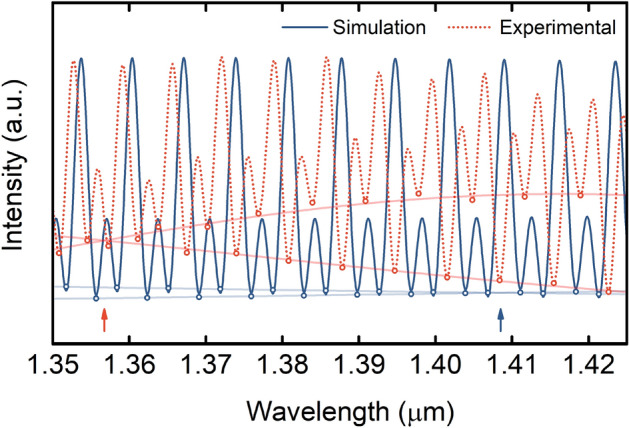
Comparison between simulated and experimental spectrum. The simulated spectrum of the structure shown in the inset of Fig. 6d, considering two modes in the sensing FPI, is represented in blue. The experimental spectrum of the structure from Fig. 7a with a few-mode sensing FPI is shown with a dotted orange line. The intersections between internal envelopes are marked with an arrow.

Figure 10 shows the superposition between the simulated and the experimental spectrum. The simulated spectrum considers the structure with the approximation of a two-mode sensing FPI, while the experimental spectrum results from a structure with a few-mode sensing FPI. The shape and visibility of the two spectra are different due to additional attenuation and imperfections in the experimental structure, not accurately accounted in the simulations. Moreover, the phase in both cases is slightly different. In one hand, the geometrical parameters used in the simulations can be slightly different from the real ones. Since the structure is highly sensitive, a small deviation in the parameters used for simulations can have a high impact in the phase of the Vernier envelopes and in the overall simulated spectrum. On the other hand, the experimental structure may present additional modes than the two considered for simulations, which contribute to an additional phase change and also to a different shape of the spectrum.

### Reproducibility of the few-mode sensing FPI

**Figure 11:**
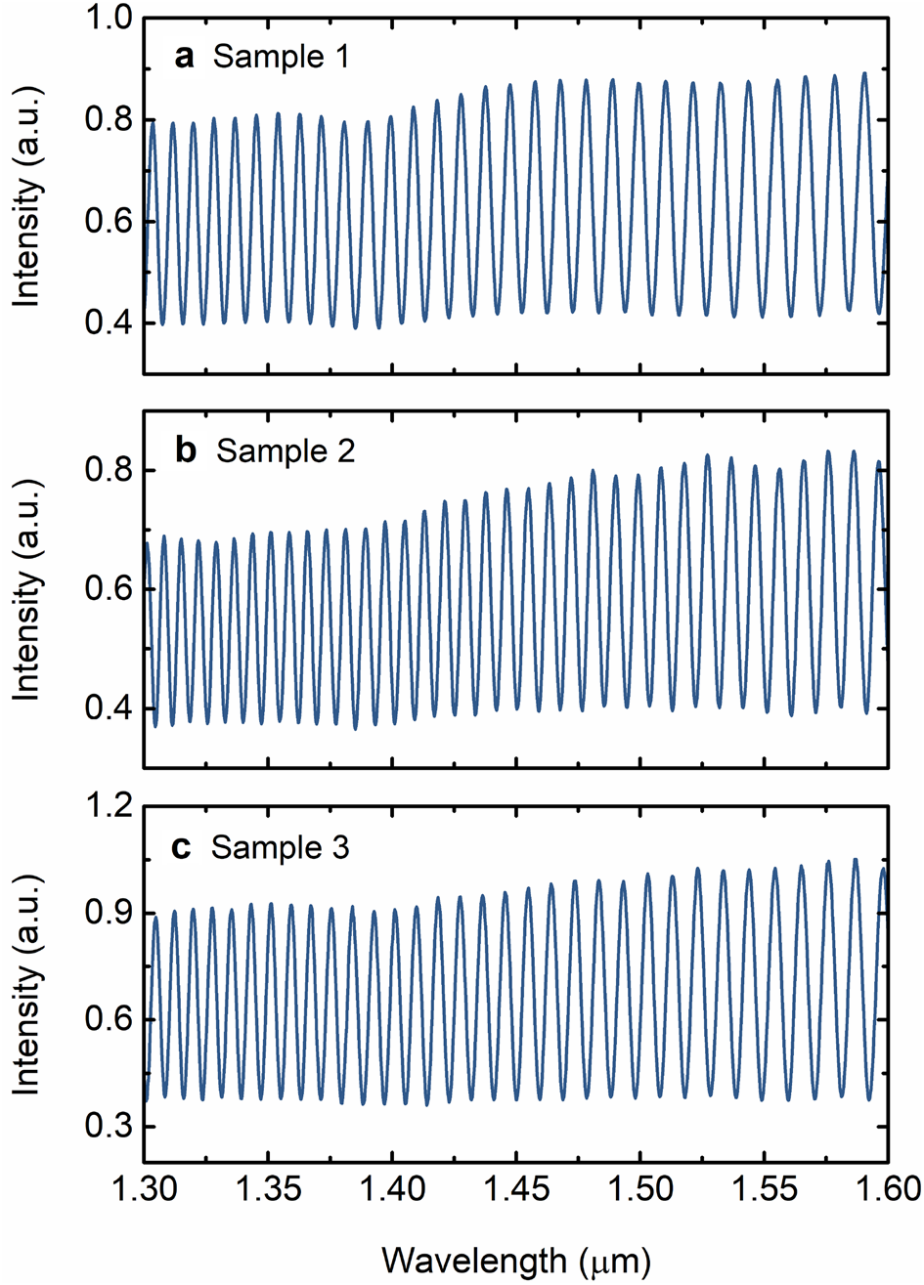
Intensity spectra of few-mode sensing FPIs. (**a**) Sample 1 corresponds to the few-mode sensing FPI used in the experiment. (**b**) and (**c**) are two additional samples fabricated using the same procedures as for sample 1, demonstrating the reproducibility of the fabrication method. The output spectra present a slight low-frequency modulation with visibility increasing towards longer wavelengths.

Figure 11 shows the output spectra of three few-mode sensing FPI samples fabricated using the procedures described in “Methods”. Sample 1, whose output spectrum is depicted in Fig. 11a, corresponds to the few-mode sensing FPI used in the experiment to produce the giant sensitivity refractometer. The other two samples, presented in Fig. 11b,c, demonstrate the reproducibility of the output spectra for different samples.
